# Preparation and Characterization of Whey Protein Isolate–DIM Nanoparticles

**DOI:** 10.3390/ijms20163917

**Published:** 2019-08-12

**Authors:** Abbas Khan, Cuina Wang, Xiaomeng Sun, Adam Killpartrick, Mingruo Guo

**Affiliations:** 1 Key Laboratory of Dairy Science, Northeast Agriculture University, Harbin, Heilongjiang 150030, China; 2 Department of Food Science, College of Food Science and Engineering, Jilin University, Changchun 30062, China; 3 Department of Food Science and Engineering, Northeast Agriculture University, Harbin, Heilongjiang 150030, China; 4 Food Science Corporation, Inc. Williston, VT 05495, USA; 5 College of Agriculture and Life Sciences, The University of Vermont, Burlington, VT 05405, USA

**Keywords:** 3,3’-diindolylmethane, whey protein isolate, nanoparticles, encapsulation

## Abstract

3,3’-Diindolylmethane (DIM) is a bioactive compound found in Cruciferous vegetables that possesses health benefits such as antioxidant, anticancer, and anti-inflammatory effects. However, hydrophobicity and photolabile limit its pharmaceutical applications. This study aims to prepare and characterize DIM-encapsulated whey protein isolate (WPI) nanoparticles mixed at different ratios of WPI and DIM using the combined heating–ultrasound method. Results showed that all the samples showed adequate physicochemical characteristics: The mean particle size of the nanoparticles could be controlled down to 96–157 nm depending on the DIM to WPI ratio used in the preparation with a low polydispersity index (<0.5), higher negative values of zeta potential (>−40 mV) as well as with greater encapsulation efficiency (>82%). Flow behavior indices showed the shear-thinning Non-Newtonian or pseudoplastic (*n* < 1) behavior of the nanoparticles. The thermal properties were characterized by differential scanning calorimetry (DSC), which showed that DIM was successfully entrapped in WPI nanoparticles. The secondary structure of WPI was changed after DIM incorporation; electrostatic interaction and hydrogen bonding were major facilitating forces for nanoparticles formation, confirmed by Fourier Transform Infrared Spectroscopy (FT-IR). Transmission electron microscopy (TEM) micrographs showed that all the samples had a smooth surface and spherical structure. The wall material (WPI) and encapsulation method provide effective protection to DIM against UV light and a broad range of physiologically relevant pH’s (2.5, 3.5, 4.5, 5.5, and 7). In conclusion, whey protein isolate (WPI)-based nanoparticles are a promising approach to encapsulate DIM and overcome its physicochemical limitations with improved stability.

## 1. Introduction

3,3’-Diindolylmethane (DIM) is a hydrophobic phytochemical, mainly found in cruciferous vegetables (such as cauliflower, Brussels sprouts, broccoli, kale, and collard greens) [1,2]. Exhaustive scientific research in the past decades on DIM has revealed that it has a broad range of biological and pharmacological activities, such as antioxidant, anti-inflammatory, antibiotic, anticancer, and other desirable medicinal benefits [3,4]. Recently, DIM has drawn more attention because of their therapeutic properties to protect and treat different types of cancers [5], especially prostate and breast cancers [6,7]. Also, the Quantitative Structure–Active Relationships (QSAR) model predicted the anticancer activity of DIM, in which eleven DIM derivatives were synthesized, two derivatives possessed potent radical scavenging activities, and some showed inhibitory activities in a primary anticancer assay in vitro. This 3D-QSAR technique is very fast and precise in analyzing and predicting the biological activity of bioactive compounds (antibiotic, antidepressant, etc.) taking in account the possible changes in the molecular structures and chromatographic parameters [8,9,10,11]. In vitro studies demonstrated that DIM has anti-proliferative and anti-cancer activities in various cancer cells, for example, breast, prostate, colorectal, endometrial, and pancreatic cancers [12,13]. Therefore, there is a need for development of a pharmaceutical DIM dosage for prevention or treatment of various diseases using DIM as an active substance. However, clinical study results reported on DIM were affected mainly because of low bioavailability, resulting from various factors, e.g., photosensitivity, limited solubility in water, and physicochemical instability [14,15]. Moreover, the use of DIM in liquid food is difficult, because of their hydrophobic nature and sensitivity to high temperature, UV light, and pH [14,16]. These properties largely limited their applications in the pharmaceutical and food industries.

In this context, nanoparticles-based microencapsulation is a promising approach to protect the hydrophobic bioactive compounds against harmful conditions. Several safe procedures were used for the production of nanoparticles in food and pharmaceutical industries from natural materials to encapsulate sensitive hydrophobic components [17,18,19]. Due to the good emulsification, solubility, and film forming properties, milk proteins are drawing increasing attention as a wall material to encapsulate many bioactive compounds [20,21,22]. Milk proteins are categorized into two main groups: Whey proteins and caseins [23]. In whey proteins, whey protein isolate (WPI) is an important by-product obtained from cheese with a protein content higher than 90%, and has been widely used as carrier materials for encapsulation due to the good emulsification and superior gelling properties [17,24]. In addition, whey protein isolate has a high nutritional quality, and is highly bioavailable with a high concentration of essential amino acids [24,25]. The main components of WPI are β-lactoglobulin and α-lactalbumin, which have excellent protective film-forming abilities (wall system) [23,26]. Whey protein isolate (WPI) may also be formed into nanoparticles. A nanoparticles-based delivery system has been used as promising material to microencapsulate various bioactive/hydrophobic compounds, such as vitamins, carotenoids, and flavor compounds [17], with improved stability and physicochemical properties [24,27,28]. The emulsifying properties of whey protein isolate could be enhanced by thermal treatment (heating above 60 °C), which has been used as an encapsulating material with improved physicochemical properties [17,28]. In order to enhance DIM physicochemical properties, very few studies have been reported. For example, the preparation of zein/carboxymethyl-based DIM nanoparticles by combined ionic gelation and the liquid–liquid phase separation method [15], encapsulation of DIM in polymerized whey protein concentrate (PWPC) [14], and development of nanocapsules for DIM encapsulation with different compositions of polymers, ethylcellulose, and oils [16]. However, to date, whey protein isolate (WPI) nanoparticles had never been employed as a wall material for microencapsulation of DIM.

Thus, this study aimed for the preparation and characterization of WPI-based DIM nanoparticles with different formulations. Different DIM ratios were used with whey protein isolate (WPI) nanoparticles to prepare a stable formulation, and were assessed regarding physicochemical, thermal, rheological, and micro-structural properties. Further, the nanoparticles were examined for pH stability and photostability.

## 2. Results and Discussion

Whey protein isolate (WPI) is considered as a promising approach to developed nanoparticles for microencapsulation of various bioactive compounds. Previously, whey protein isolate nanoparticles have been developed in our laboratory to protect and improve physicochemical, microstructural, and rheological properties of many hydrophobic compounds, e.g., astaxanthin and totarol [24,28]. In the current study, whey protein isolate nanoparticles were developed to encapsulate DIM in order to improve the physicochemical properties and provide effectual protection during storage. For this purpose, four formulations were prepared with different WPI/DIM ratios, ranging from 2.5:1 to 10:1, using a high intensity ultrasound for 5 min, and were subsequently assessed for physicochemical characterization.

### 2.1. Physicochemical Determination of DIM-Encapsulated WPI Nanoparticles

**Figure 1:**
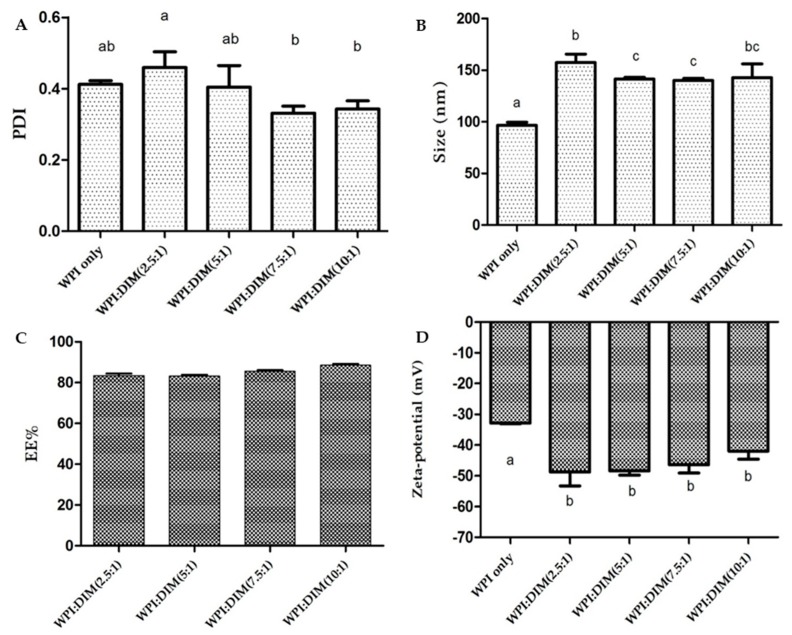
(**A**) Polydispersity indexes (PDI), (**B**) particle size, (**C**) encapsulation efficiency (EE%), and (**D**) zeta potential of whey protein isolate only and diindolylmethane-encapsulated WPI nanoparticles. Values with different letters represent a significant difference (*p* < 0.05).

Particle size and zeta potential are important properties to provide valuable information of micro-encapsulated compounds regarding the formation of stable formulations. The particle size of WPI-only and DIM-encapsulated whey protein nanoparticles treated by ultrasound are shown in Figure 1. The mean particle size values of DIM-encapsulated whey protein isolate nanoparticles were 157.47 ± 8.09, 141.43 ± 1.62, 140.00 ± 2.16, and 142.83 ± 13.23 nm for samples with WPI to DIM ratios of 2.5:1, 5:1, 7.5:1, and 10:1, respectively, which were found to be significantly higher as compared to native WPI (96.53 ± 3.02 nm) (*p* < 0.05). This increase in the particle size might be due to the presence of DIM entrapped in or absorbed on β-lactoglobulin (the main component of whey protein isolate) due to an enhanced hydrophobic interaction. β-lactoglobulin can bind hydrophobic compounds by their three binding sites. At pH 7.0 the loop EF (strands) is open, which allow ligands to enter into the hydrophobic core and can bind the large numbers of small hydrophobic molecules causing increases in particles size [29,30]. Moreover, these results suggested that WPI/DIM nanoparticle size was dependent on the addition of DIM to WPI nanoparticles (core-coating ratios), and increased by increasing the DIM content in the system. [17,31,32]. Moreover, a small droplet size was obtained for all formulations with WPI nanoparticles, which reduced gravity force and helped the system to remain stable with no proof of separation, coalescence, or flocculation [33]. These results were supported by the TEM images (Figure 5). In addition, DIM is a hydrophobic compound and interacts with proteins through a strong hydrophobic interaction, which results in a more compact structure in nanoparticles [15,30]. Nanoparticles prepared with WPI exhibited a lower particle size compared to other proteins due to their good emulsification property, which acts as a surfactant because of their hydrophilic and hydrophobic regions [24,34,35].

The zeta potential values of DIM-encapsulated WPI nanoparticles are presented in Figure 1D. The zeta potential value of WPI-only (−32.86 ± 0.20 mV) significantly dropped to (−42.00 ± 2.62)–(−48.76 ± 4.55) mV after DIM was coated by WPI nanoparticles (*p* < 0.05), indicating that DIM maybe attached to WPI nanoparticles. Similar results with reduction in zeta potential values after encapsulation were showed by Luo et al. who reported that zeta potential value was reduced from −11.2 and −9.98 mV to −20 mV after encapsulation of DIM with zein/CMCS [15], and addition of DIM in β-lactoglobulin decreased the zeta potential value from −8.63 to −17.93 mV [30]. Furthermore, increasing the DIM ratio (WPI to DIM ratios ranging from 1:10 to 1:2.5) showed no significant impact (*p* < 0.05) on zeta potential of the nanoparticles, which is suggesting that the large amount of DIM entrapped in the core has not affected the surface charge of the β-lactoglobulin. Zeta potentials of all samples were highly negative, which lead to more repulsive forces between particles and could enhance the physical stability of various systems [36]. The particle size of WPI-only was less than 100 nm. However, WPI:DIM nanoparticle particle size was in the range of 96–157 nm, which could be counted as nanoparticles [14,15].

Figure 1A shows the polydispersity indexes (PDI) values of all formulations. Nanoparticles with greater PDI values suggest a broad size distribution, which suggest its more susceptibility toward Ostwald ripening. However, a lower PDI value suggests a relative narrow distribution with good physical stability [30,37]. Compared with WPI-only, the PDI value was increased when DIM was incorporated in WPI nanoparticles with a volume ratio of 2.5:1 (WPI:DIM), followed by a decrease in PDI value with a higher concentration of DIM. However, the changes were not significant (*p* < 0.05). The decreased PDI value at higher DIM concentration may be due to the more interacted WPI and DIM, which became more uniform with a narrow size distribution [24]. Moreover, the polydispersity indexes (PDI) values of all the formulations were less than 0.4 except for the WPI:DIM ratio (2.5:1) (Figure 1A), suggesting narrow distribution with a good stability.

Figure 1C shows the encapsulation efficiency of DIM dispersed in WPI nanoparticles. It was observed that encapsulation efficiency was decreased with an increase in DIM content to WPI nanoparticles, suggesting that a small amount of DIM was not entrapped in the protein matrix instead of being embedded. This characteristic was also found by Patel et al. who revealed that EE decreased with increasing curcumin concentration in the protein nanoparticles, which led to precipitation [31]. All the samples’ encapsulation efficiency was above 80%. A similar observation was reported in a previous study showing that the encapsulation efficiency (EE) of astaxanthin encapsulated with whey protein isolate were greater than 90% for all samples [24].

### 2.2. Thermal Properties of the DIM-Encapsulated WPI Nanoparticles

**Figure 2:**
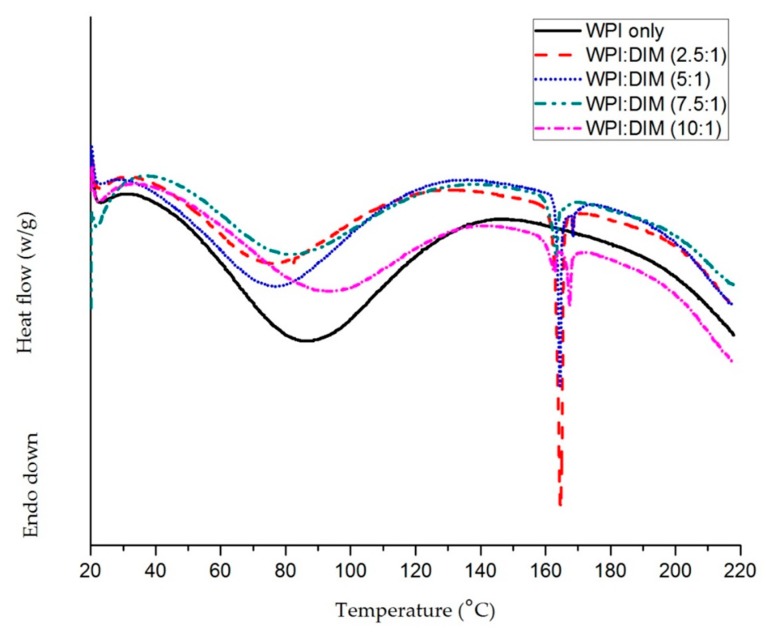
Differential scanning calorimeter (DSC) curves of WPI and DIM-encapsulated WPI nanoparticles.

Figure 2 shows the DSC thermograph of the freeze-dried whey protein isolate–DIM nanoparticles and the native WPI sample, over a broad temperature range (20–220 °C). The DSC curve of WPI-only showed a broad endothermic peak between 40 and 115°C, centered at 87 °C. This characteristic endotherm is attributed to heat-induced transitions occurring in the main constituent of whey protein(β-lactoglobulin and α-lactalbumin). Moreover, the endothermic peak of the physical mixture of WPI and DIM (i.e., WPI+DIM with different ratios) existed between 70 and 100 °C (Figure 2), which might be related to evaporation of absorbed water. A similar characteristic was also noticed in previous literatures [24,38]. Sharp peaks were observed in all formulations of WPI–DIM nanoparticles approximately at 165 °C, which is considered as the melting point. However, no melting point was investigated in native WPI. For the physical mixture of WPI and DIM nanoparticles, both the endothermic peak of WPI and the melting peak of DIM has been detected (Figure 2). However, only an endothermic peak was observed in the WPI-only sample, which suggested clear evidence that DIM was molecularly dispersed in the polymeric matrix and hence properly encapsulated in WPI nanoparticles. Similar observations were reported where both endothermic and melting point peaks were detected after VD3 encapsulation with protein nanoparticles, which gave evidence of nanoencapsulation [38,39].

### 2.3. Rheological Properties of the DIM-Encapsulated WPI Nanoparticles

**Figure 3:**
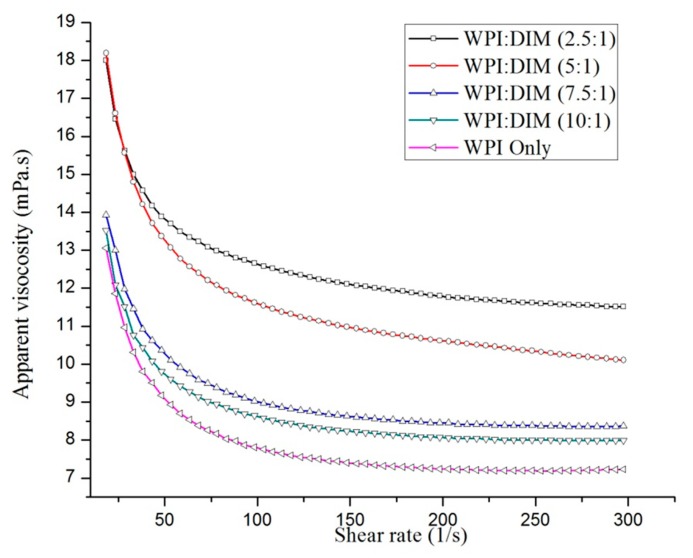
Flow behavior of WPI-only and DIM-encapsulated WPI nanoparticles.

The apparent viscosity as a function of the shear rate of WPI-only and DIM-encapsulated whey protein isolate nanoparticles mixed with different ratios are shown in Figure 3. All samples’ flow behavior indices showed shear-thinning Non-Newtonian or pseudoplastic (*n* < 1) behavior, decreasing apparent viscosity with an increase in shear rate followed by a constant viscosity at a higher shear rate. This is because all the large particles and flocs decreased in size and only small particles remained [40]. This characteristic behavior was defined as all the nanoparticles had identical apparent viscosity, due to the particles having the same degree of aggregation [41]. On the other hand, the increasing DIM ratio with WPI nanoparticles had a significant effect (*p* < 0.05) on the viscosity of DIM-encapsulated WPI nanoparticles. By looking at these flow curves, a clear increase in the nanoparticles viscosity was observed by increasing the DIM ratio from 10:1 to 2.5:1, followed by constant viscosity at a higher shear rate (Figure 3) [14]. Hydrocolloid addition could increase the viscosity of the nanoparticles aqueous phase and can lead to reducing the mean particle size of the dispersed phase, which is related to the increased viscosity of the whole system [42]. WPI-only and WPI:DIM mixed at the 10:1 or 7.5:1 ratio showed prominent shear-thinning behavior at the lower shear rates region, while WPI DIM (5:1 or 2.5:1) viscosities declined very slowly in all shear rate (γ) ranges. Previous researches claimed similar interesting flow behavior in hydrocolloid dispersions [43,44]. This flow behavior of DIM-encapsulated WPI fitted well (*R*^2^ ≥0.997 in all cases) with the Sisko model [45]:

*ɳ_app_* = *ɳ_∞_*+ *k*_ᴑ_γ^n−1^

where *ɳ_app_* denotes the apparent viscosity (Pa s), *ɳ_∞_* and *k*_ᴑ_ are the infinite shear rate viscosity and the consistency index, respectively, γ is the shear rate (s^−1^), and *n* is the flow index [40].

In the current study, the log *ɳ_app_* vs. log γ results is well explained by the Sisko model, and was clearly presented (Figure 3). In addition, all the sample viscosities were in the range of 6–11 mPa s; pseudo-plastic behavior was reflected in all cases as parameter (*n*) was found less than one (*n* < 1). It was observed that increasing the DIM content to WPI tend to increase parameter (*ɳ_∞_*) (Table 1). Rheological data of various emulsions and dispersions also reported the same shear-thinning flow behavior, and the same model was used [30,46].

### 2.4. Fourier Transform Infrared Spectroscopy (FT-IR)

**Figure 4:**
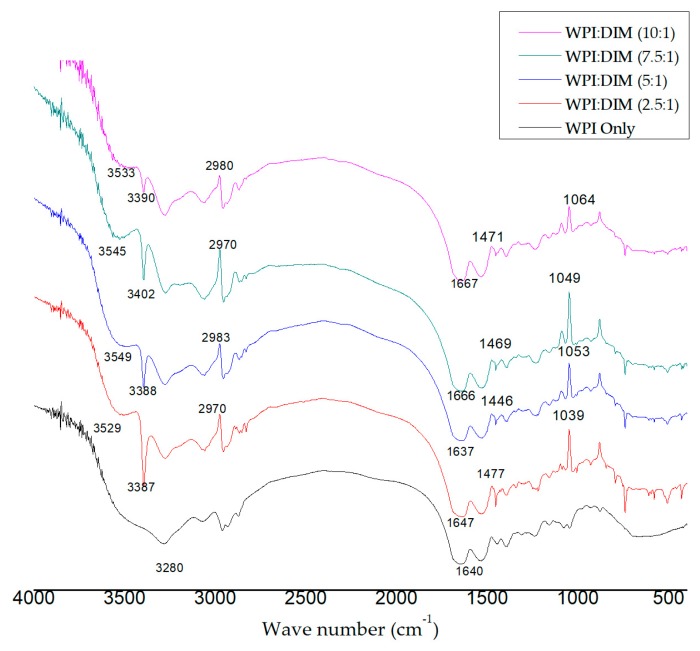
Fourier transform infrared spectroscopy (FT-IR) spectra of whey protein isolate and diindolylmethane-encapsulated whey protein isolate nanoparticles with different ratios.

Figure 4 shows the FT-IR spectra of WPI-only and WPI–DIM nanoparticles, which showed the interaction of WPI and DIM. In the infrared spectra, interesting peaks were observed in the range of 3200–3600 cm^−1^, indicating hydrogen bonding. A strong peak appeared at the spectrum of WPI-only at 3280 cm^−1^ attributed to O–H stretching of hydrogen bonding [47]. However, this O–H stretching (3280 cm^−1^) was remarkably shifted to 3387, 3388, 3402, and 3390 cm^−1^ afterDIM was encapsulated in WPI nanoparticles due to the interaction of DIM and WPI. These changes suggested that hydrogen bonding was formed between β-lactoglobulin and DIM. These findings are parallel with previous research for the development of DIM-based polymerized whey protein nanoparticles [14]. In addition, major characteristic peaks of O–H-free hydroxyl group were detected in the range of 3500–3600 cm^−1^ in the spectra of encapsulated DIM, showing more hydrogen bonding were formed, which is considered to be the major facilitating force for nanoparticles formation [48]. Hydrogen bonding and hydrophobic interaction maybe the main forces related to the interaction of whey protein and hydrophobic substances [30]. Several characteristic peaks due to C–H stretching of the aliphatic group were observed at 2970, 2983, 2970, and 2980 cm^−1^ in the spectra of microencapsulated DIM with different ratios [14,49]. Amid I and Amid II bands are considered as the backbone of whey protein isolate, confirming the presence of α-lactalbumin and β-lactoglobulin, which are the main constituents of whey protein isolate. The Amid I observed at range of 1600–1700 cm^−1^ in the encapsulated DIM are mainly assigned to the C=O stretching vibration carbonyls group. Amid II absorption bands displayed at 1446, 14469, and 1471 cm^−1^, respectively, correlate to bending group of N–H [35]. By comparing the spectrum of WPI with encapsulated DIM, peaks of Amid I were shifted from 1640 cm^−1^ to 1647, 1666, and 1667 cm^−1^, with different ratios of DIM in WPI, indicating the presence of an electrostatic interaction between WPI and DIM rather than the chemical reactions. Therefore, it is concluded that the interaction of WPI and DIM is based on electrostatic forces [49]. An increase in the intensity of Amid I and Amid II also suggests the binding of DIM to β-lactoglobulin; similar results were revealed by Wang et al. [30]. Changes in the intensity suggests the hydrophobic interaction of DIM and β-lactoglobulin complexes.

### 2.5. Transmission Electron Microscopy (TEM)

**Figure 5:**
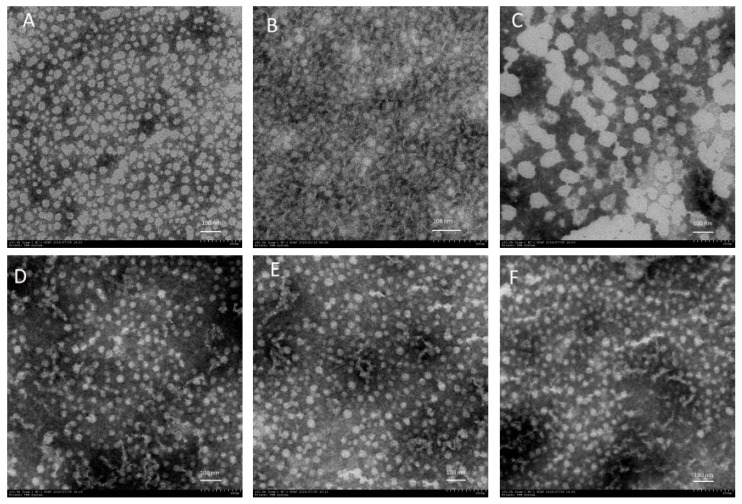
Transmission electron microscopy of native diindolylmethane, native whey protein isolate, and DIM encapsulated with WPI nanoparticles.(**A**) Diindolylmethane; (**B**) Whey protein isolate only; (**C**)WPI:DIM (2.5:1); (**D**) WPI:DIM (5:1); (**E**) WPI:DIM (7.5:1); and (**F**) WPI:DIM (10:1).

The micrograph of the WPI-only and DIM-encapsulated WPI nanoparticles were investigated using transmission electron microscopy (TEM) (Figure 5). The nanoparticle micrographs illustrated remarkable differences depending on DIM mass ratios in the WPI system. It is clear that an increase in the DIM ratio with WPI resulted in larger particle sizes; these results are consisted with dynamic light scattering DLS data (Figure 1). The WPI-only micrograph showed a polymeric network (unfolding of protein particles occurred) due to thermal treatment. The particle size was also found to be smaller compared to encapsulated DIM nanoparticles with different ratios (Figure 5). However, in general, the nanoparticles images with different ratios (Figure 5B–D) showed a smaller size as compared to DLS data, because the zetasizer results were the hydrodynamic diameter of the particles with a layer of water, however the samples examined by TEM was air dried and water was removed. On the other hand, the native DIM micrograph exhibited a rough-edge morphology [14]. All the formulations of DIM-encapsulated WPI nanoparticles with different WPI:DIM ratios (5:1, 7.5:1, and 10:1) showed a smooth, spherical, and more apparent shape after encapsulation [14,47]. However, WPI:DIM mixed with the 2.5:1 ratio was observed with a greater particle size covered by a dense–rigid polymeric network of WPI, with no porous structure. These results coincide with the DLS data (Figure 1). This difference in structure maybe due to the increased hydrophobic and electrostatic interaction [50,51].

### 2.6. Photo-Chemical Stability

**Figure 6:**
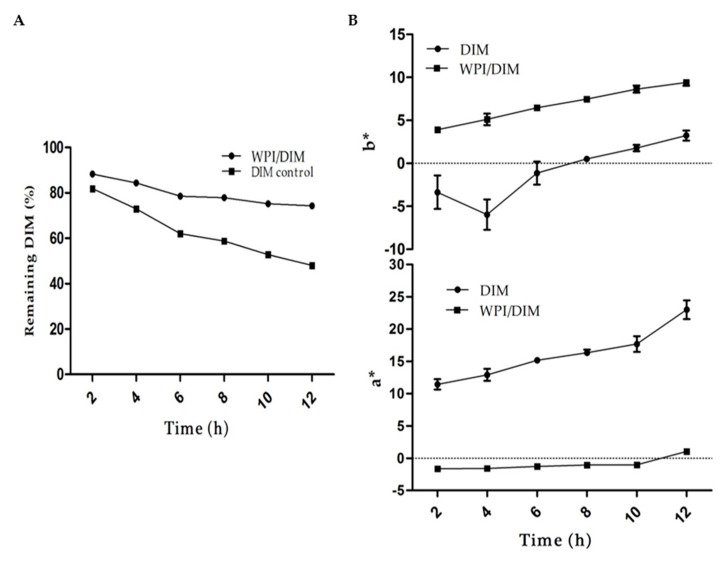
Photochemical stability of (**A**) native DIM and (**B**) WPI:DIM (10:1) samples against UV light during storage for 12 h. Values are means ± SD (*n* = 3). a*-values referred to redness/greenness (higher positive value means redness); and b*-values referred to yellowness/blueness (higher positive value means yellowness).

Light is an important factor causing isomerization, oxidation, and oligomerization of many phytochemicals. DIM when exposed to UV light may result in instability, because of their aromatic ring which possesses UV absorption ability [52]. The results from UV light are shown in Figure 6. During 12 h of UV irradiation, DIM in whey protein isolate nanoparticles was observed more stable as compared to the control sample (DIM only). The results showed that WPI nanoparticles provide better protection to DIM, as more than 70% of DIM remained unchangeable in WPI nanoparticles. However, only 48% DIM were found at the end of 12 h in the control DIM. Encapsulation of DIM in protein nanoparticles is a proper approach to protect it from harsh conditions. Previous researches demonstrated that incorporating DIM into nanocapsules protected DIM from light-induced changes and increased scavenger activity [16]. DIM was encapsulated in protein nanoparticles, and more than 60% of DIM was detected during incubation for 4 h when exposed to UV light [15].

The light and heat may cause color changes and chemical degradation to many bioactive compounds such as carotenoids and phytochemicals. The native DIM showed redness in color when exposed to heat or light upon incubation due to oxidation, oligomerization, and isomerization [14,52]. The effect of UV light on WPI with and without DIM is shown in Figure 6. Results showed a rapid increase in color intensity (a*-value) in pure DIM as compared to encapsulated DIM with WPI nanoparticles upon incubation for 12 h, suggesting that DIM was properly entrapped in WPI nanoparticles. This increase in the redness attributed to the fact that more light was absorbed by pure DIM as compared to encapsulated DIM, which caused chemical degradation [16]. The b*-value of WPI/DIM and native DIM followed an almost similar trend upon exposure of UV light. The b*-value of both samples was increased slightly throughout incubation. This change in b*-value in WPI/DIM nanoparticles could be attributed to the color changes of wall material as well, as previous study revealed that whey protein might undergo slight color changes upon heating [53]. These monitored changes in the color values (a*- and b*-value) of the samples may therefore be taken as overall color changes of the nanoparticles upon exposure to UV light.

### 2.7. Impact of pH

**Figure 7:**
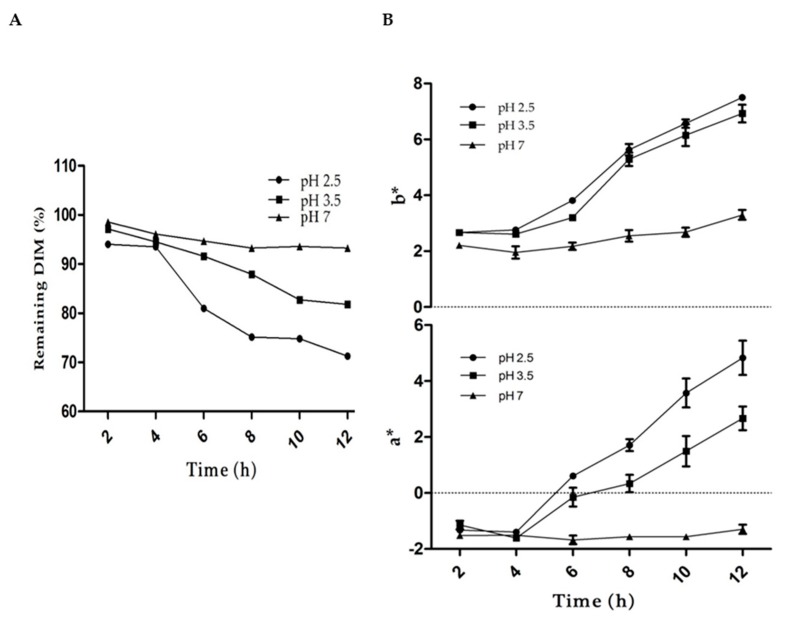
Effect of pH on (**A**) DIM and (**B**) color change (a*-values and b*-values) of WPI:DIM (10:1) during storage for 12 h. Values are means ± SD (*n* = 3).

The effect of pH on color and DIM loss was studied (Figure 7). At pH 4.5 and 5.5, which is in proximity to the isoelectric point of whey protein, the samples were totally unstable to droplet, creaming, and aggregation. The results showed that the sample at pH 7 was more stable and less changes occurred in the color (a*- and b*-values) or DIM loss as compared to pH 2.5 or 3.5. However, the overall visual appearance of sample showed no clear change in color at any pH values. Previous research reported that phytochemicals and carotenoids in acidic environments results in degradation and isomerization due to the protonation of the carbon atoms of the conjugated system [54,55]. The higher stability of the DIM in WPI nanoparticles could be attributed to the presence of hydroxyl groups, which limits the protonation [53]. Moreover, microencapsulation of phytochemicals in protein nanoparticles is considered as a good vehicle to protect it from harsh environments [14,15].

## 3. Materials and Methods

### 3.1. Materials and Reagents

Whey protein isolate (WPI) powder containing 93.14% protein was supplied by Hilmar (Turlock, CA, USA). 3,3′-Diindolylmethane was provided by Luotian Xinpusheng Pharmaceutical Co., Ltd. (Hubei, China). Deionized Milli-Q pure water was taken from a Milli-Q water filtration system (Millipore Corp., Bedford, MA, USA).

### 3.2. Nanoparticles Preparation

#### 3.2.1. Aqueous Phase

An 8% whey protein isolate (WPI) solution (*w*/*v*) was developed with deionized Milli-Q water and continuously stirred (1500 rpm) at room temperature for 1.5 h as an aqueous phase. Then, the solution was refrigerated for 24 h for complete hydration. The solution was adjusted by 1 M NaOH solution to pH 7.0, followed by heating to 80 °C for 15 min with constant mechanical stirring to denature WPI, and then equilibrated at ambient temperature.

#### 3.2.2. Organic Phase

The DIM was dispersed in pure ethanol (15 mg/mL) to prepare a stock solution, according to the previous method explained by Khan et al. with some modifications [14]. Then the diindolylmethane solution was heated to 40 °C to achieve a clear solution (i.e., with no visible DIM in solution) under mild stirring for 2 h.

#### 3.2.3. Preparation of DIM-Loaded WPI Nanoparticles

DIM-loaded WPI nanoparticles were prepared by mixing the organic phase in theaqueous phase indifferent ratios, i.e., WPI:DIM ranging from 2.5:1 to 10:1 (*v*/*v*) to obtained desired ratios, with continuous stirring for 2 h. Then the mixture was homogenized for 5 min with high intensity ultrasound at an amplitude of 30% in an ice bath using a VCX800 (Vibra Cell, Sonics, Newtown, CT, USA) with a 13mm diameter probe (high-grade titanium alloy).

### 3.3. Determination of Particle Size, Polydispersityindex (PDI),and Zeta Potential

Characterization of nanoparticles in terms of mean particle size (Z-average), PDI, and zeta potential was assessed using a Zetasizer Nano ZS 90 (Malvern Instruments, Worcestershire, UK). Fresh nanoparticle suspensions were used for analysis of nanoparticles characterization. Briefly, samples were diluted to (0.01%) with deionized water, and 1 mL of each sample was poured into measuring cell for analysis. All the samples were analyzed for three independent measurements at 25 °C.

### 3.4. Encapsulation Efficiency (EE)

EE was measured according to the method as explained with some modifications [24]. To assess the EE, DIM was extracted from the WPI–DIM nanoparticles suspension using DMSO and methanol. Briefly, 2 mL of the sample was mixed with 4 mL of DMSO/methanol (2:2, *v*/*v*), vortexed for 10 min, and centrifuged at 2200g for 10 min. The DIM in the upper supernatant was transferred to a 10 mL brown volumetric flask. The extraction was repeated 3 times with 2 mL DMSO until the aqueous layer was clear and then measured with a UV-visible spectrophotometer (UV-2550, Shimadzu) at 281 nm using an established standard curve (*R*^2^ = 9966). The EE was calculated as follows:

$$EE\%=\frac{\mathrm{Total}\;\mathrm{DIM}\;\mathrm{amount}-\mathrm{Free}\;\mathrm{DIM}\;\mathrm{amount}}{\mathrm{Total}\;\mathrm{DIM}\;\mathrm{amount}}.$$

### 3.5. Differential Scanning Calorimetry (DSC)

Thermal properties of freeze-dried WPI-only and WPI–DIM nanoparticles (pre-frozen at −80 °C overnight followed by drying at 4 °C for 24 h at 0.3MPa) were performed using TA instruments (New Castle, DE, USA). A small amount of 5–10 mg of the sample was put in aluminum pans and sealed hermetically; a blank pan was run as a reference. The temperature rose from room temperature to 20–220°C.

### 3.6. Determination of Rheological Properties

A parallel plate geometry (60mm diameter; 1 mm thickness; gap width of 1000 µm) was used to determine rheological properties of the nanoparticles suspension using a Hybrid Rheometer (TA Instruments, New Castle, DE, USA) attached with a system (Thermo Cube, NY, USA) for cooling. The apparent viscosity of the WPI-only and WPI-based DIM nanoparticles was recorded as a function of shear rate.

Samples flow ramp was assessed in the shear rate range of 0.1 to 300 s^−1^ at 25 °C.

Samples peak holds were analyzed at 200 s^−1^ for 60 s at 25°C.

### 3.7. Fourier Transform Infrared Spectroscopy (FT-IR)

The chemical structure of nanoparticles was observed using an FT-IR spectrophotometer (IR-Prestige 21, Shimadzu, Kyoto, Japan). The KBr-pallet method was used to prepare the samples for analysis. Briefly, the solid sample (2.0 mg) was obtained by freezing the sample at −80 °C overnight followed by drying for 24 h at 0.3 MPa and then mixed with native potassium bromide (KBr) powders (200 mg), and ground to a fine powder. The spectra were recorded in the range between 500 and 4500 cm^−1^ wavelengths. Pure KBr powder was run as a blank.

### 3.8. Transmission Electron Microscopy (TEM) Analysis

TEM was used to observe the microstructural properties of the nanoparticles. Micrographs of the nanoparticle suspensions composed of different DIM ratios were observed using transmission electronic microscopy (H-7650, Hitachi High-Technologies, Tokyo, Japan). All the samples were diluted to (0.1%) using ultra deionized water. Then, a small droplet (~10 µL) was placed onto a carbon-coated copper grid. The samples were air dried and photographs were taken at different magnifications. 

### 3.9. The Influence of Encapsulation on Stability of DIM

The photostability and pH stability of DIM-encapsulated WPI nanoparticles and native DIM were determined for color changes and DIM content in WPI nanoparticles as a function of time. For this purpose, a freshly prepared sample(WPI:DIM with a volume ratio 10:1) as explained in Section 3.2.3 with DIM dispersion in ethanol (15 mg/mL) as a control in a UV-transparent glass vial (20 mL)was exposed from both sides to two UV light bulbs (20 W)for 12 h in a lightproof cabinet. To measure the DIM content, DIM was extracted after every 2 h by diluting 1 mL of the sample with 2 mL DMSO/methanol (1:1, *v*/*v*), vortexed for 10 min and centrifuged at 2200g for 10 min. The DIM in the upper supernatant was transferred and measured with a UV-visible spectrophotometer (UV-2550, Shimadzu) at 281 nm. Color changes were recorded using a hand-held colorimeter (CM 2300d, Konica Minolta, Osaka, Japan) by measuring (a*-value, b*-value) color coordinates. a*-values referred to redness/greenness (higher positive value means redness, higher negative value means greenness); and b*-values referred to yellowness/blueness (higher positive value means yellowness, higher negative value means blueness) [52]. For the pH stability test, all the pH samples were adjusted to desired pH values (2.5, 3.5, 4.5, 5.5, and 7) using either a 2 M HCL or 2 M NaOH solution. Then, samples were transferred into test tubes, and were stored at ambient temperature for 12 h. Then, color changes and DIM content were measured as described above. Each sample was measured in triplicate.

### 3.10. Experimental Design

All the experiments were carried out in triplicate. All the data presented are in mean ± standard deviation. Statistics analyses were conducted using SPSS 20.0 (SPSS Inc. Chicago, IL, 184 USA). The resulted data were subjected to analysis of variance (*p* < 0.05) and comparisons of means were performed by Tukey‘s test.

## 4. Conclusions

DIM-encapsulated whey protein isolate (WPI) nanoparticles were successfully developed, using the combined heating–ultrasound method. All the formulations showed smaller particles sizes (96–157 nm) with a higher encapsulation efficiency (>82%). The interaction of DIM and WPI was evidenced by DSC and FT-IR data. The TEM analysis showed that the nanoparticles had a smooth surface and spherical shape. Whey protein isolate nanoparticles were able to enhance DIM photochemical and pH stability when compared with native DIM. In conclusion, a WPI-based DIM nanoparticles approach and the encapsulation method used in this study provided effectual protection during storage with improved physicochemical characteristics. These characteristics made WPI nanoparticles an attractive wall material for the encapsulation of bioactive components in the development of nutraceutical and food products.

## Figures and Tables

**Table 1:** Infinite-shear-rate viscosity, consistency index, and flow index.

Samples	*ɳ_∞_* (mPa∙s) Infinite-Shear-Rate Viscosity	*k*_ᴑ_ Consistency Index	*n* Flow Index
WPI only	6.74 ± 0.02^a^	137.940 ± 0.007^a^	−0.049 ± 0.019^a^
WPI:DIM (2.5:1)	10.82 ± 0.04^b^	75.743 ± 0.003^b^	0.180 ± 0.015^b^
WPI:DIM (5:1)	9.17 ± 0.03^c^	85.891± 0.002^c^	0.223 ± 0.008^c^
WPI:DIM (7.5:1)	7.87 ± 0.02^d^	109.056 ± 0.005^d^	0.018 ± 0.016^d^
WPI:DIM (10:1)	7.58 ± 0.01^e^	119.131 ± 0.004^e^	−0.030 ± 0.013^e^

WPI: Whey protein isolate; DIM: Diindolylmethane. Values with different letters in the same column denote a significant difference (*p* < 0.05).
